# Engineering a Vascularized Hypoxic Tumor Model for Therapeutic Assessment

**DOI:** 10.3390/cells10092201

**Published:** 2021-08-26

**Authors:** Yuta Ando, Jeong Min Oh, Winfield Zhao, Madeleine Tran, Keyue Shen

**Affiliations:** 1 Department of Biomedical Engineering, Viterbi School of Engineering, University of Southern California, Los Angeles, CA 90089, USA; yutaando@usc.edu (Y.A.); ohjeongm@usc.edu (J.M.O.); wtzhao@usc.edu (W.Z.); madeleit@usc.edu (M.T.); 2 Norris Comprehensive Cancer Center, Keck School of Medicine, University of Southern California, Los Angeles, CA 90033, USA; 3 USC Stem Cell, Keck School of Medicine, University of Southern California, Los Angeles, CA 90033, USA

**Keywords:** vasculature, angiogenesis, tumor microenvironment, hypoxia, viscous fingering

## Abstract

Solid tumors in advanced cancer often feature a structurally and functionally abnormal vasculature through tumor angiogenesis, which contributes to cancer progression, metastasis, and therapeutic resistances. Hypoxia is considered a major driver of angiogenesis in tumor microenvironments. However, there remains a lack of in vitro models that recapitulate both the vasculature and hypoxia in the same model with physiological resemblance to the tumor microenvironment, while allowing for high-content spatiotemporal analyses for mechanistic studies and therapeutic evaluations. We have previously constructed a hypoxia microdevice that utilizes the metabolism of cancer cells to generate an oxygen gradient in the cancer cell layer as seen in solid tumor sections. Here, we have engineered a new composite microdevice-microfluidics platform that recapitulates a vascularized hypoxic tumor. Endothelial cells were seeded in a collagen channel formed by viscous fingering, to generate a rounded vascular lumen surrounding a hypoxic tumor section composed of cancer cells embedded in a 3-D hydrogel extracellular matrix. We demonstrated that the new device can be used with microscopy-based high-content analyses to track the vascular phenotypes, morphology, and sprouting into the hypoxic tumor section over a 7-day culture, as well as the response to different cancer/stromal cells. We further evaluated the integrity/leakiness of the vascular lumen in molecular delivery, and the potential of the platform to study the movement/trafficking of therapeutic immune cells. Therefore, our new platform can be used as a model for understanding tumor angiogenesis and therapeutic delivery/efficacy in vascularized hypoxic tumors.

## 1. Introduction

A characteristic feature of solid tumors in advanced cancers is the complex yet functionally abnormal network of blood vessels formed through tumor angiogenesis, which is considered one of the hallmarks of cancer [1]. In healthy tissues, angiogenesis is delicately balanced by pro- and anti-angiogenic factors [2]. In tumors, the balance is shifted toward a chronic pro-angiogenic state, largely driven by the state of oxygen deficiency or hypoxia in cancer cells [3,4]. Hypoxia modulates vascular endothelial growth factor (VEGF) to promote angiogenesis [5] while inducing vascular permeability [6,7], resulting in leaky blood vessels that support tumor growth and facilitate metastatic dissemination [8]. The vascular defects also impede the delivery of cancer therapeutics in the tumor microenvironment (TME), such as small molecule inhibitors, antibodies, and therapeutic immune cells [9]. For cell-based immunotherapies, the endothelium further forms a physical barrier through downregulation of molecules crucial for immune cell adhesion [10,11] or upregulation of proteins that suppress trans-endothelial migration [12]. Endothelial cells may also directly regulate the anti-tumor immune responses [13]. However, despite these advances, it remains difficult to study the cellular mechanisms of tumor-vasculature interplay or to evaluate the therapeutic efficacies of drugs and cell-based immunotherapies in such complex microenvironments in vivo.

Microengineered in vitro models, also known as the microphysiological models, have emerged as powerful tools to assess the biological states and therapeutic responses in various tissues and organs [14,15]. Recently, vascularized 3-D microengineered in vitro tumor models have been developed to recapitulate the TME [16,17,18], investigate intravasation [19,20], and predict therapeutic outcomes in vivo [21]. The 3-D architecture of such tumor models captures the branching morphogenesis [22] and transitional phenotypes which are distinct from traditional 2-D cultures [23]. On the other hand, unlike the conventional spheroids, bulk hydrogel, or scaffold-based 3-D culture models [24,25], these microengineered platforms allow for precise spatial control of stromal components and physiological mimicry of tumor vasculature while maintaining the compatibility with high-content imaging modalities. However, existing bioengineered tumor models often fail to recapitulate a key feature of the TME, i.e., a hypoxic gradient in the tumor bulk interacting with a 3-D vasculature. Efforts have been made by exposing the vascularized microtumor models in uniform hypoxic incubators [26], which misses the gradient of oxygen and the resultant metabolic heterogeneity in cancer cells in vivo. This is mainly due to the materials and fabrication approaches used in the existing microfluidic vascular models, which remain largely restricted to the highly oxygen-permeable silicone materials and soft lithography/replica molding processes.

In this study, we built a bioengineered vascularized tumor model which interfaces a microfluidic 3-D microvasculature with a 3-D hypoxic tumor model [27]. We demonstrated the capacity of the platform to capture the progression of angiogenesis at a micron-scale spatial resolution over a week. Furthermore, the vasculature allows investigation of the delivery of chemicals and cell-based therapy. Our composite microdevice-microfluidics platform is a versatile tool for gaining rapid insights into the interactions of multiple cell types in the TME, and for assessing the efficacy and safety of therapeutics.

## 2. Materials and Methods

### 2.1. Cell Culture

SKOV3 human ovarian cancer cells were obtained from ATCC and maintained in Dulbecco’s modified Eagle’s medium (DMEM) supplemented with 10% fetal bovine serum (FBS; Sigma-Aldrich, St. Louis, MO, USA) and 100 U/mL penicillin and 100 µg/mL streptomycin (P/S, Thermo Fisher, Waltham, MA, USA). Panc1 human pancreatic cancer cells and MDA-MB-231 human breast cancer cells were also obtained from ATCC and maintained in Rosewell Park Memorial Institute (RPMI) 1640 medium supplemented with 10% FBS and P/S. Primary human bone marrow stromal cells (BMSCs) derived from whole human bone marrow aspirates (Lonza, Basel, Switzerland) were cultured in the MesenCult Proliferation Kit (Stem Cell Technologies, Vancouver, Canada). Human umbilical vein endothelial cells (HUVECs) were obtained from Lonza and maintained in the endothelial cell growth medium-2 (EGM-2) BulletKit (Lonza, Basel, Switzerland). HUVECs expressing a red fluorescence protein (RFP) reporter gene were obtained from Angio-Proteomie. Cells from 75–90% confluent monolayers were passaged using 1% trypsin in phosphate-buffered saline (PBS, Thermo Fisher, Waltham, MA, USA).

### 2.2. 3-D Micropatterning and Hypoxic Tumor Model and Oxygen Level Measurement

**Figure 1:**
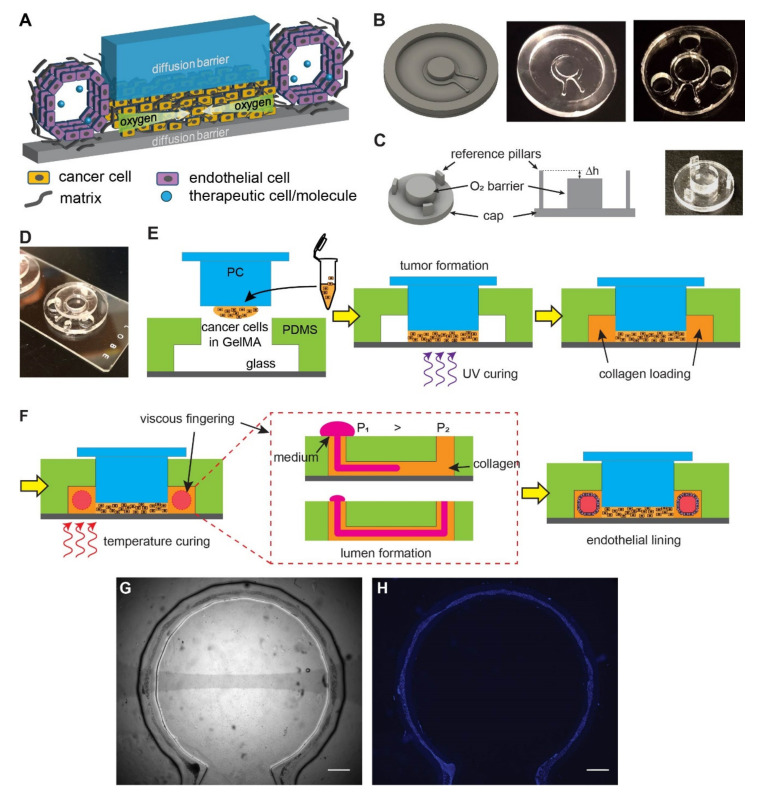
Design and fabrication of the vascularized hypoxic tumor model. (**A**) Schematics of hypoxia device with surrounding vasculature. (**B**) Design of the microfluidic mold, the milled polycarbonate (PC) product, and the replica molded PDMS device (with punched holes for the reference pillars). (**C**) Design of the oxygen barrier cap, the role of reference pillars in determining the gap size (Δh), and the milled PC cap. The Δh determines the thickness of tumor section in the model. (**D**) An assembled device on a glass slide. (**E**) The assembly of the tumor model prior to vascular formation. (**F**) Formation of vasculature by viscous fingering and endothelial lining. (**G**) A completed device with endothelial lining in bright field. (**H**) Fluorescent nuclear staining of the endothelial lining (blue) for the device in (**G**). Scale bars: 1 mm.

The design and toolpaths for the hypoxia microdevice were created in Autodesk Fusion360 (Autodesk, Inc., San Rafael, CA, USA). The oxygen diffusion barrier cap consists of an oxygen barrier pillar (6 mm diameter) 100 µm shorter than the three reference pillars, which determine the gap size for oxygen diffusion and hydrogel height [28]. A master mold was milled to produce a PDMS fluidic chamber and channel through replica molding, which fit with the hypoxia cap (Figure 1B). The PDMS chamber and channel device is plasma-bonded onto a clean glass slide, which is then assembled with the hypoxia cap (Figure 1D). Both the hypoxia cap and the master mold for the PDMS device were milled in polycarbonate (PC). Immediately before 3-D micropatterning, the PDMS channel was coated with polydopamine (PDA), as previously described [29]. Briefly, 2 mg/mL dopamine hydrochloride solution (*w*/*v* in 10 mM Tris HCl buffer, pH 8.5) was introduced to the PDMS channels and incubated for 2 h at room temperature, followed by extensive rinsing with H_2_O and drying. Cancer cells were resuspended in gelatin methacryloyl (GelMA) and 10 × DMEM (prepared from powder DMEM; Thermo Fisher, Waltham, MA, USA) to achieve a cell density of 10 million cells/mL in 10% GelMA and 1 × DMEM. The final pH of the cell-laden solution was adjusted to approximately 7, as determined by a pH indicator strip. This mixture was pipetted onto the oxygen barrier pillar, before it was assembled into the PDMS chamber/channel device, to form the tumor layer by the surface tension between the glass slide and the oxygen barrier pillar. Next, the platform was exposed to UV light for 120 s to crosslink the GelMA. The UV power was measured as 2.34 ± 0.26 mW/cm^2^ by a handheld radiometer (Solarmeter Model 5.0; Solar Light, Glenside, PA, USA).

Oxygen levels were measured using fluorophore-based microparticle sensors [27,28]. Silica gel particles with 10–14 µm diameter were preactivated with 0.1 N NaOH and incubated with 0.5 mM tris (4,7-diphenyl-1,10-phenanthroline) ruthenium (II) dichloride (Thermo Fisher, Waltham, MA, USA) in ethanol for 30 min. The particles were then washed 3 times with deionized water and then once with ethanol before being dried in a 70 °C oven overnight. The dried silica gel was then dispersed and mixed in Sylgard 184 PDMS (mixed at 10:1 base to curing agent ratio). A drop of the mixture was added and spread on the milled PC cap by another milled mold with a complementary concave shape and cured overnight. The cap was then used in the same way as a non-coated PC cap to form tumor section and imaged in Acridine Orange channel (Ex: 480/30 nm, Em: 620/60 nm) to indicate spatial distribution of oxygen levels.

### 2.3. Patterning an Artificial Vasculature

Vascular lumen was created using the viscous fingering method, as previously described [16,30,31]. Briefly, 0.5 mg/mL fibronectin (Sigma-Aldrich, St. Louis, MO, USA) was introduced to the fluidic channel after 3-D micropatterning of cancer cells, and incubated for 20 min. After aspirating the fibronectin solution, 3 mg/mL rat tail collagen type I (Corning, Corning, NY, USA) was loaded and incubated at 37 °C for 2 min to partially polymerize and increase the viscosity of the collagen solution [32]. Next, a droplet of cell culture media was passively pumped onto the inlet of the PDMS channel. Cell culture media, due to its lower viscosity, displaced the center of the more viscous collagen hydrogel, resulting in a lumen through the hydrogel along the fluidic channel. The microdevice was incubated at 37 °C for 20 min to complete the polymerization of collagen.

To line the lumen with endothelial cells, HUVECs were introduced to the lumen at 40,000 cells/µL. The microdevice platform was taped to a rod attached to a motor (BBQ Rotisserie Variable Speed Reversible Brushless Gear Motor; Wondermotor, CA, USA), and incubated at 37 °C at 2 RPM for 30 min. The device was further incubated for 2 h with no rotation. Then, fresh media were perfused through the channel to rinse unattached cells. Vascular structure was assessed over 1 week in the microdevice.

### 2.4. COMSOL Multiphysics^®^ Modeling

Expected oxygen gradients across the 3-D cell bulk were simulated with COMSOL Multiphysics modeling software (COMSOL; Burlington, MA, USA). Passive oxygen diffusion within the media was assumed to be governed by the generic diffusion equation of gas in water [33], with a diffusion coefficient of 3 × 10^−9^ m^2^/s. Boundary conditions were approximated so that the microdevice was impermeable to oxygen; the media surface in contact with atmosphere had a fixed oxygen concentration corresponding to the normoxic level (0.2 mol/m^3^). Cellular density was assumed to be homogeneous throughout the hydrogel. Cellular oxygen consumption was assumed to follow Michaelis–Menten kinetics with a logistic function constraining consumption below a critical oxygen level:

(1) $$R_{O_{2}}=R_{max}\left(\frac{C}{C+k_{MM,O_{2}}}\right)\cdot \delta \left(C>C_{cr}\right)$$

where $R_{max}$ is the maximum oxygen consumption rate of cancer cells adjusted for their average cell volume (0.02 mol s^−1^ m^−3^) [33,34], $k_{MM,O_{2}}$ is the Michaelis–Menten constant corresponding to the oxygen concentration where consumption is half maximal, $C_{cr}$ is the critical oxygen concentration below which necrosis is assumed to happen and cells cease oxygen consumption, and δ is the step-down function accounting for the termination of oxygen consumption [33]. The step-down function was COMSOL’s smoothed Heaviside function with a continuous first derivative and no overshoot (flc1hs in COMSOL Multiphysics). Cell number in the 3-D micropattern was then incorporated into the total oxygen consumption rate of the 3-D bulk. All geometries in the model were defined with an extremely fine mesh in COMSOL Multiphysics.

### 2.5. Immunostaining

After 24, 48, or 72 h of incubation, samples were fixed in 4% paraformaldehyde (PFA), permeabilized with 0.1% Triton X-100 (Fisher Scientific, Waltham, MA, USA), blocked for 3 h with 4% bovine serum albumin (GE Healthcare Bio-Sciences, Chicago, IL, USA), incubated in primary (overnight) and secondary antibody (1 h), and mounted with FluoroGel II containing 4′,6-diamidino-2-phenylindole (DAPI) (Electron Microscopy Sciences, Hatfield, PA, USA) on glass slides. Primary antibodies used were anti-Glucose Transporter 1 (Glut-1) (ab15309, 1:200) (Abcam, Cambridge, MA, USA) and common endothelial markers such as anti-CD31 (89C2, 1:1000) (Cell Signaling Technology, Danvers, MA, USA) and VE-cadherin (2158, 1:100) (Cell Signaling Technology, Danvers, MA, USA) [35,36]. With the immunostained samples, a Nikon Eclipse Ti inverted fluorescent microscope was used to acquire the wide-field epifluorescence images, while a Nikon C2 confocal microscope was used to acquire z-stacks of fluorescence images for 3-D reconstruction and analysis.

### 2.6. Image Analysis

Images were analyzed using ImageJ (NIH, Bethesda, MD, USA) and Imaris (Bitplane AG, Zurich, Switzerland) software. To determine the diameter of the engineered vasculature over time, the length from the two vasculature walls perpendicular to the direction of the vasculature was measured. The Imaris software was used for 3-D reconstruction of confocal image slices. All image figures shown were representative of ≥3 independent runs/repeats.

### 2.7. Statistics

Data were presented in mean ± S.D and statistical analyses were performed using GraphPad Prism 9 (GraphPad Software, Inc, San Diego, CA, USA). One-way ANOVA or two-tailed Student’s *t*-tests were used for evaluating the significance of difference unless otherwise indicated. N.s.: not significant (*p* > 0.05), *: *p* < 0.05.

## 3. Results

### 3.1. Micromilling and Viscous Fingering Enable a Composite Vascularized Hypoxic Tumor Model

We designed a vascularized hypoxic tumor model containing two major elements: a hypoxic tumor section [27,28] and an endothelialized microchannel surrounding the tumor section (Figure 1A). The hypoxic tumor section is composed of cancer cells embedded in a layer of extracellular matrix (ECM), which is sandwiched between two oxygen diffusion barriers (blue and gray pieces in Figure 1A). The oxygen consumption by cell metabolism, combined with the limited oxygen supply by diffusion from the edge, results in a lateral, centripetal oxygen gradient in the artificial tumor section, as seen in tumors in vivo [27,28]. The endothelialized microchannel mimics the blood vessels surrounding tumor nests in real tumors, which allows for the study of angiogenesis in response to tumor hypoxia, as well as the delivery of oxygenated medium, nutrients, drugs, and/or therapeutic cells (Figure 1A).

To achieve this design, we fabricated the microfluidic channel and the top oxygen diffusion barrier in two separate processes. Micromilling was chosen in both processes to fabricate the microfluidic mold and the diffusion barrier (Figure 1B,C). It is a computer-numerical-controlled (CNC) machining process with micrometer precision that allows for rapid prototyping without the need for cleanroom facilities, while having high special resolution as well as flexibility in the material choices (such as those with low oxygen permeability such as polycarbonate (PC), used in this study) [37]. The microfluidic channel was replica-molded in the highly oxygen-permeable polydimethylsiloxane (PDMS) from a micromilled PC mold (Figure 1B). The oxygen barrier cap was directly milled in PC for its low oxygen permeability. It contains a central oxygen barrier pillar and three reference pillars (Figure 1C). The reference pillars were designed to achieve and maintain the height of the tumor section (Δh) within the hypoxic tumor model. Before assembly, the PDMS device was punched with three holes to accommodate the oxygen diffusion barrier cap (Figure 1B). The PDMS microfluidic channel was then bonded onto a glass slide by oxygen plasma treatment and inserted with the oxygen diffusion barrier cap in the final assembled device (Figure 1D, without cells).

The workflow of the tumor model assembly is illustrated in Figure 1E,F. A dense ECM network and heterogeneous oxygen levels are major hallmarks of solid tumors. To recapitulate these factors, cancer cells were embedded in gelatin methacryloyl (GelMA), a photocrosslinkable hydrogel derived from natural collagen [38,39]. A mixture of cancer cells with 10% GelMA (10 million cells/mL) was cured with ultraviolet (UV) light (λ = 375 ± 14 nm) (Figure 1E), which resulted in a hydrogel stiffness at approximately 6 kPa in Young’s modulus, resembling typical cancer matrix stiffnesses [40]. The artificial vasculature was engineered in a two-step process. First, a lumen was generated in the collagen-filled fluidics channel with viscous fingering patterning [30] (Figure 1F). Viscous fingering is a phenomenon where a less viscous fluid displaces a more viscous fluid, resulting in projections of the less viscous fluid (Figure 1F, middle). Upon gelation of collagen at the 37 °C physiological temperature, a hollow lumen remains. Next, the lumen was seeded with human umbilical vein endothelial cells (HUVECs) while the device was rotated continuously. As a result, the entire lumen became lined with endothelial cells that mimic a blood vessel (Figure 1G,H).

### 3.2. An Artificial Vasculature Can Be Formed and Maintained in the Hypoxic Tumor Model

**Figure 2:**
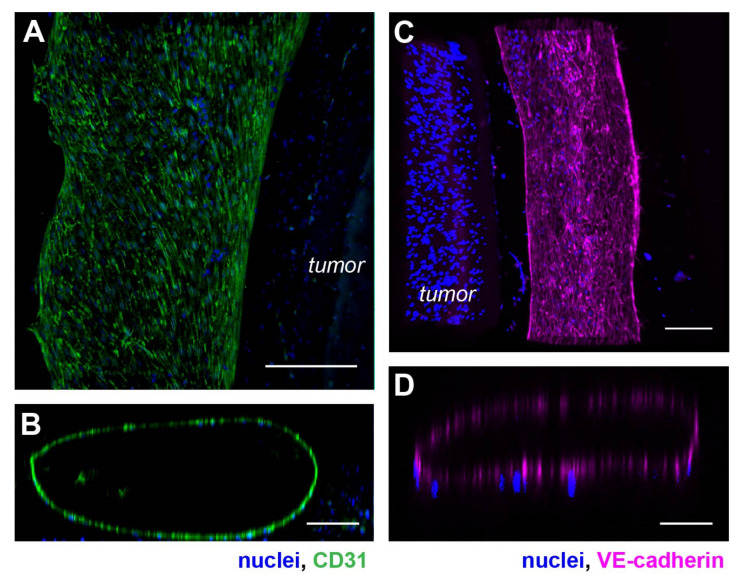
Phenotypic characterization of the vasculature in hypoxic SKOV3 tumor model. (**A**) Top view of a 3-D vasculature surrounding a tumor section reconstructed from confocal scans after CD31 immunostaining in HUVEC. Blue: nuclei. (**B**) A vertical volume-rendered cross section reconstructed from confocal images showing a hollow lumen surrounded by an endothelial layer. (**C**) Top view of a 3-D vasculature surrounding a tumor section reconstructed from confocal scans after immunostaining of VE-cadherin in the tumor model with nuclear counterstain. (**D**) A vertical, volume-rendered section reconstructed from VE-cadherin confocal scans. Scale bars: (**A**,**C**) 250 µm; (**B**,**D**) 100 µm.

**Figure 3:**
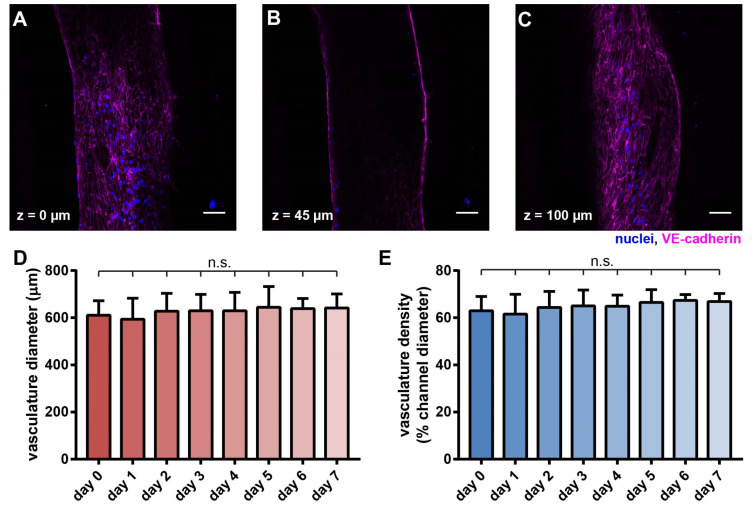
Morphological characterization of the vasculature in the hypoxic tumor model. Confocal scans of the vascular cross-sections at (**A**) z = 0 µm, (**B**) z = 45 µm, and (**C**) z = 100 µm, respectively. Scale bars: 50 µm. (**D**) Vascular diameter and (**E**) vascular density were maintained over 7 days. (**D**,**E**) *n* = 12, 14, 15, 15, 9, 9, 6, 6 for day 0~7, respectively. N.s.: not significant, with one-way ANOVA.

Next, we examined the phenotypic and morphological characteristics of the vasculature formed in the device. We first imaged the structure of the engineered vasculature with immunostaining against vascular biomarkers. We used HUVEC cells to line a collagen lumen formed around a hypoxic tumor section composed of SKOV3 ovarian cancer cells in the model. We then fixed the cells and immunostained for CD31 (also known as the platelet-endothelial cell adhesion molecule-1 or PECAM-1), to highlight the vascular morphology and cell–cell adhesion [41]. With confocal microscopy, we visualized the vasculature with maximum intensity projection at the lateral dimensions (Figure 2A) and 3-D reconstruction of a vertical cross-section (Figure 2B). We found that the HUVEC formed a monolayer along the surface of the collagen lumen in the microfluidic channel. Interestingly, these endothelial cells spontaneously formed an aligned pattern on the vascular surface, with their long axes pointing toward the longitudinal direction of the vascular lumen (Figure 2A).

We also stained the vasculature for the vascular endothelial-cadherin (VE-cadherin). VE-cadherin is an endothelial-specific adhesion molecule expressed at the cell–cell junctions in vasculature, which controls vascular permeability and leukocyte extravasation [42]. Similar analyses of maximum intensity projection of a section of the vasculature showed a tube-like structure lined with HUVECs (Figure 2C,D). Compared to the CD31 staining, VE-cadherin was visually more specifically localized to the cell–cell junctions, as expected with strong cell-cell interaction in the HUVEC monolayer of the vasculature wall (Figure 2C). A vertical confocal section of the vasculature also confirmed a hollow lumen (Figure 2D).

With 3-D sectioning in confocal microscopy, we found that HUVECs uniformly adhered to the lumen wall, as shown in confocal slices of the vasculature at the indicated heights (Figure 3A–C). The size of the vasculature was consistently approximately 600 µm wide and 100 µm tall. Notably, the collagen channel contained geometrical irregularities due to the interfacial instability during the viscous fingering process [43]. In the formed vascular lumen, some regions in the confocal slices appeared to be darker than the surrounding regions (Figure 3A,C), which were due to the difference in focus and contrast caused by the irregular wall shapes in the continuous endothelial layer (Supplemental Figure S1). A common issue in using collagen as a hydrogel for cell embedment is compaction [29,44]. To investigate the effect of collagen compaction in our model, we monitored the vasculature daily for a week. Collagen compaction visibly occurred and led to the vasculature collapsing when the PDMS fluidic device/channel was not coated with polydopamine (PDA) [29] and fibronectin [45] (Supplemental Figure S2).

In contrast, upon surface coating with PDA and fibronectin, the collagen compaction was prevented, and the structural integrity of the vasculature was well maintained over 7 days without any noticeable decay (Figure 3D,E).

### 3.3. A Hypoxic Gradient Can Be Established in a 3-D Tumor Section with Surrounding Vasculature

**Figure 4:**
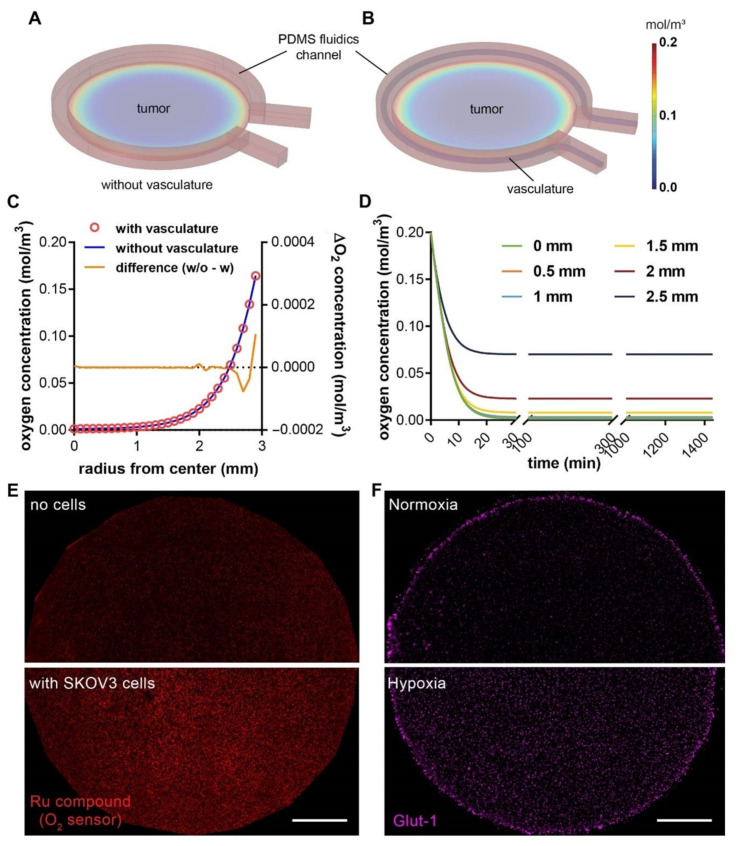
COMSOL simulation of oxygen profiles in hypoxic tumor models without or with the vascular channel. Oxygen profile in (**A**) a tumor model with microfluidic channel alone, and (**B**) a composite vascularized hypoxic tumor model. (**C**) Radial distribution of oxygen concentrations from the center to the edge of the tumor region without (blue line; the left y-axis) or with vasculature (red circles; the left y-axis), and the difference between the two (orange line; the right y-axis). (**D**) Rapid achievement of the steady-state oxygen levels at different radial positions in the vascularized tumor model. Model dimensions are the same as the actual sizes. (**E**) Measurement of oxygen levels in the tumor section without (**top**) or with (**bottom**) SKOV3 cancer cells at 24 h, using an oxygen-sensitive ruthenium compound absorbed in silica microparticles and embedded in a thin layer of PDMS on the PC pillar. Higher fluorescence indicates lower oxygen level. (**F**) The expression of Glut-1, a hypoxic marker, in the embedded SKOV3 cancer cells in the tumor section in normoxia (**top**, hypoxia model removed of the PC cap on day 0) vs. hypoxia (**bottom**) model after 24 h of culture. Scale bars: 1 mm.

We next investigated whether the new vascularized design of the hypoxic tumor model would alter the oxygen profile that has been previously characterized in the non-vascularized model [27]. We carried out computer simulations with COMSOL Multiphysics^®^ to characterize the spatial distribution and temporal evolvement of oxygen concentration in the 3-D microdevice platform (Figure 4A,B). We found that, with or without the endothelial component in the device, the oxygen gradients follow very similar spatial patterns in both tumor models, with near anoxia at the center and steep gradient close to the periphery (Figure 4C). In the absence of the vasculature, the oxygen level was slightly higher at the immediate edge of the tumor section, whereas the centers of the tumor sections were minimally affected by the vascularization (Figure 4C, yellow curve). This is due to the fact that endothelial cells predominantly undergo glycolytic metabolism, which consumes minimal oxygen and allows for transferring as much oxygen as possible through the endothelium to the perivascular tissues [46]. On the other hand, similar to our previous reports [27,28], the induction of hypoxia in the central tumor section happens in a very rapid manner, which reaches >90% of the steady-state oxygen levels within the first 30 min (Figure 4D). We further confirmed the hypoxic state of cancer cells within the tumor section, indicated by the elevated fluorescence of the fluorescent oxygen sensor particles compared to the no-cell control (Figure 4E), as well as the enhanced expression of glucose transporter-1 (Glut-1) in the embedded SKOV3 cancer cells compared to the normoxic counterpart (Figure 4F). The results here indicate that the characterization of the oxygen profiles in our previously reported 3-D hypoxic tumor model still applies to the new vascularized models in this study, to guide the investigation of hypoxia-induced angiogenesis.

### 3.4. Progression of Tumor Angiogenesis Is Observed in the Composite Tumor Model

**Figure 5:**
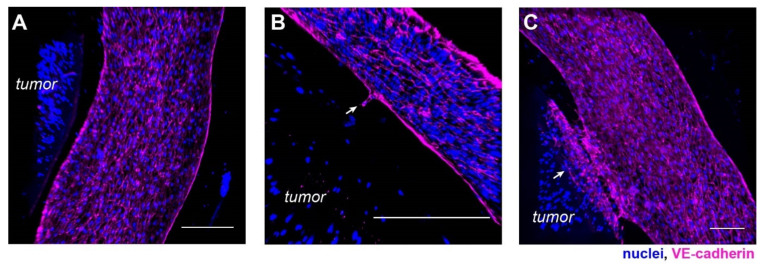
Angiogenesis in a hypoxic SKOV3 tumor model. (**A**) A vascular segment without angiogenic activities in early culture. (**B**) Vascular sprouting toward the tumor section on day 5. (**C**) Massive endothelial ingrowth into the tumor section in one vascular segment on day 7. Scale bars: 250 µm.

Tumor angiogenesis results from the complex crosstalk between tumor cells and the surrounding vasculature in the TME over time. An important advantage of our platform is the ability to capture micro-scale processes with high-content imaging. We reconstructed the structure of the endothelial lumen from confocal images with the Imaris software, to assess the angiogenic progression in our solid tumor model composed of SKOV3 cells (Figure 5A–C). Angiogenic activities were absent in most vascular segments within the SKOV3 model in the early cultures (Figure 5A). Interestingly, we started to observe angiogenic stalk formation around day 5, which pointed towards the hypoxic tumor section (Figure 5B). By day 7, some vascular sections had massive endothelial ingrowth into the tumor section (Figure 5C). However, such extensive angiogenic projections into the tumor bulk happened rarely in this SKOV3 model, which was largely due to the proximity between the vasculature and the tumor section.

**Figure 6:**
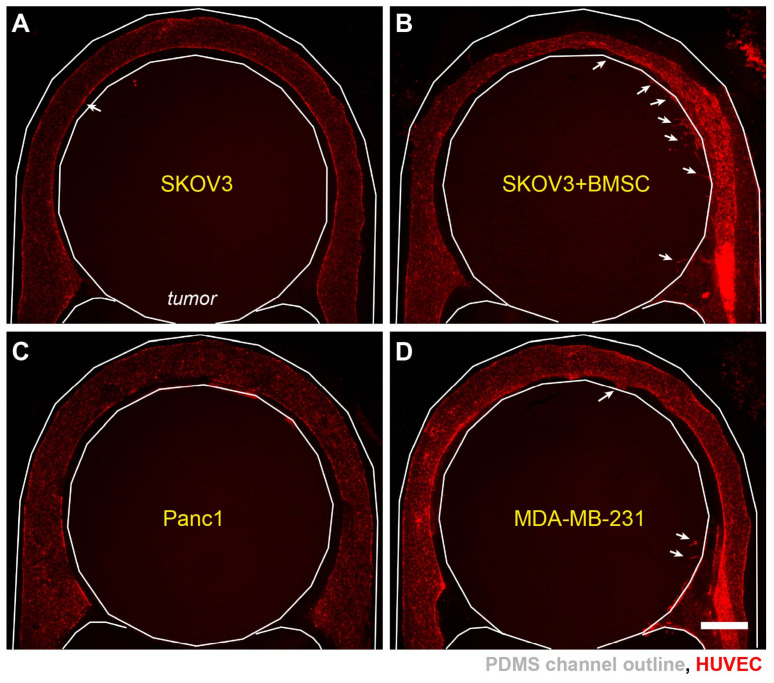
Differential angiogenic responses in different tumor models. Vascularized hypoxic tumor models on day 7 with (**A**) SKOV3 cells, (**B**) SKOV3+BMSCs, (**C**) Panc1 cells, and (**D**) MDA-MB-231 cells in the tumor sections. White arrows: incidence of vascular sprouting. Scale bars: 1 mm.

To further assess the impact of cell types in TME on angiogenesis, we set up several tumor models with different cancer cells or with/without stromal cells. It has been reported that hypoxia promotes the recruitment of bone marrow stromal cells (BMSCs) to the solid tumor microenvironment [47], which can in turn increase tumor angiogenesis [48]. We mixed BMSCs with SKOV3 cancer cells in hydrogel to form the tumor section. The angiogenic sprouting was more readily observed in the tumor sections with BMSCs than those with SKOV3 cells alone (Figure 6A,B). Interestingly, the addition of BMSCs also led to narrower vascular diameter in some areas (Figure 6B, top), suggesting a role of BMSCs in promoting the remodeling of the ECM and vascular morphologies. To investigate the effect of different cancer types in inducing angiogenic sprouting, in addition to the ovarian (SKOV3) cancer cells, we created 3-D tumor sections with pancreatic (Panc1) and breast (MDA-MB-231) cancer cells (Figure 6C,D). Surprisingly, all the models with cancer cell alone did not exhibit large number of sprouting events. Our results thus indicate a stronger role of the stromal factors in promoting angiogenesis in the hypoxic tumors.

### 3.5. Molecular and Cellular Deliveries Can Be Tracked in the Vascularized Tumor Model

**Figure 7:**
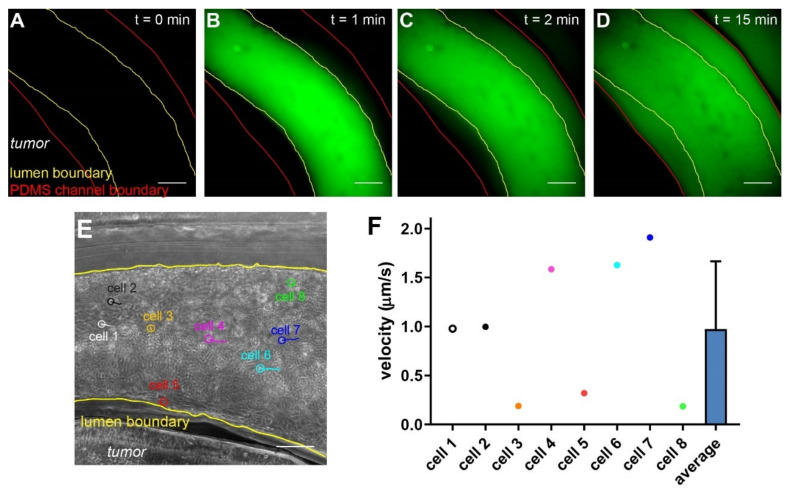
Assessing deliveries of molecules and cells in the vascularized tumor model. (**A**–**D**) Time series of the leakage of 70 kDa Dextran across the endothelium. (**E**) CAR T cells (circled) and their movement trajectories. (**F**) Velocity of CAR T cells in the vasculature (*n* = 8). Scale bars: 250 µm.

Of all the applications of vascularized in vitro tumor models, assessing the delivery of molecular and cellular therapeutics will provide insights into mechanisms of the vascular deliveries and potential interventions that can improve the therapeutic efficacies. To evaluate such potentials, we first tested the permeability of the endothelial lining in the vascularized hypoxic tumor model with 70 kDa fluorescent Dextran, which mimics the size of serum albumin (66 kDa) and is often retained in the healthy vasculature within 60 min of infusion [49]. We introduced the fluorescent Dextran from the inlet and monitored the fluorescent signal around the vasculature over time (Figure 7A–D). Upon the initial infusion, the fluorescent signal was largely retained in the vasculature (Figure 7B). It then gradually increased in the collagen matrix outside of the vasculature, often appearing on one side before extending to the other (Figure 7C,D), which indicates leakage(s) located at the side of higher initial signals in the vasculature.

Next, we evaluated the delivery of engineered T cells in our model. Chimeric antigen receptor (CAR) T cells have been heavily investigated in treating solid tumors in recent years [50]. CAR T cells have to arrest on and penetrate through the endothelium, which negatively regulate T cell functions [51], before reaching the cancer cells expressing the tumor (associated) antigens. HER2-targeting CAR T cells were introduced through the inlet into the vasculature, and their movement was tracked using microscopy (Figure 7E). With live imaging microscopy, we can analyze the speed and direction of immune cells on a single cell basis (Figure 7E,F). The majority of the infused CAR T cells remained in the center of the vasculature and moved quickly along with the flow. Notably, some T cells managed to arrest on the endothelium, as demonstrated by their minimal migration speed and short movement trajectories (Figure 7E,F, cell #3, 5, and 8). Interestingly, we noticed that some cells (Figure 7E, cell #5) were moving against the flow, albeit at low speed. This suggests an active attachment/migration of the therapeutic cells on the endothelium, which may precede their penetration through the vasculature into the tumor section.

## 4. Discussion and Conclusions

Here, we presented a composite microdevice-microfluidic platform that integrates a vascular lumen in a hypoxic tumor model. Our platform takes a combinatorial approach of micropatterning, micromilling, and microfluidic technologies to recapitulate a vascularized, hypoxic TME. Micromilling allows for choosing materials that have the desired low oxygen permeability for the hypoxia module, which is difficult to achieve with PDMS in the conventional microfluidic models. The microfluidics, on the other hand, facilitates the creation of an artificial endothelium resembling the morphology and functions of tumor vasculature. Such modular design also enables a vasculature mimetic interface without affecting the induction of an oxygen concentration gradient in the tumor section. This oxygen gradient was confirmed with the oxygen sensor layer in the device (Figure 4E). It is noteworthy that the hypoxic marker, Glut-1 did not follow a similar gradient pattern in the device (Figure 4F). It has been previously shown that, although Glut-1 is a good phenotypic marker for tumor hypoxia, Glut-1 expression correlates poorly with measured oxygen tension in patient tumors [52]. In our experience, the Glut-1 expression level in cancer cells in the 3-D model does not follow a proportional relationship with the hypoxic level in the range of oxygen levels (0~20%), and instead resembles more of an “on” switch below a certain range. Different cancer cell lines also have different radial Glut-1 patterns under similar hypoxia conditions (data not shown). Thus, we use the oxygen sensor for the oxygen levels and Glut-1 expression as confirmation of the induction of hypoxic state in the cells, to indicate both physical and biological states in the device. For vascular formation in microfluidic devices, many groups have used direct endothelial cell lining inside the PDMS channels, micro-molding or patterning in hydrogels, or spontaneous microvasculature formation through vasculogenesis/angiogenesis [18]. These methods often involve non-physiological morphology/substrate or require complex molding steps or extended culture time. The viscous fingering approach was chosen specifically for its advantage in simple and rapid fabrication, compatibility with our composite and curved microchannel design, and physiological endothelial-ECM interactions. Additional improvement on the morphology of the vascular lumen may be achieved by incorporating electrical field during viscous fingering [43].

A significant advantage of our platform is in its compatibility with high-content imaging over spatial and temporal dimensions, with its thin-layer design and lateral arrangement of cell types and tumor/vascular components. It is suited for investigating angiogenic progression around hypoxic tumors in the TME, which can provide insights into the molecular mechanisms that contribute to tumor progression. For instance, our vascularized hypoxic tumor model may be used to mimic the early tumor progression, where vasculature surrounds avascular tumor nests, and angiogenic sprouts are induced to penetrate the surrounding ECM into the overgrown, hypoxic tumor. We also showed distinct angiogenic behaviors in the vasculature without and with stromal cells. Using different cancer cells alone did not affect the frequency of angiogenesis with the cell lines we tested. It is worth noting that some vascular sprouts under cancer-cell-alone conditions were tiny (Figure 5B) and thus difficult to identify/quantify under low-magnification imaging condition for quantification (Figure 6). It is thus premature to conclude that cancer cells alone do not support angiogenesis. Additional studies with longer incubation times may be needed to further determine the angiogenic events. In contrast, co-culturing cancer cells with BMSCs drastically increased the frequency of long angiogenic sprouts and changed the vascular morphology. Our model can be further adapted to include different stromal cells, such as fibroblasts and immune cells (such as macrophages and myeloid-derived suppressor cells) [53], to understand their impact on tumor angiogenesis or elucidate their respective roles in tumor vascular invasion and metastasis under the context of tumor hypoxia.

The tumor vasculature formed in this study showed leakage in the Dextran test. It is known that tumor-vascular crosstalk results in leaky vasculature [5]. On the other hand, irregular geometric features of the vascular wall from viscous fingering (Figure 3A,C) may also have contributed to weak spots due to curvature or lower ECM density. Notably, mechanical factors such as physiological fluid flow have been shown to play a role in endothelial cell maturation and permeability [54]. It will thus be important to include the flow condition in studying tumor-vasculature interactions, the vascular leakage, as well as therapeutic deliveries (e.g., drugs and immune cells). While our artificial vasculature was cultured under static conditions and the vasculature expresses mature endothelial cell markers, it can be readily extended to include continuous fluid flow using gravitational force [55] or syringe-based pumps [56], using the existing microfluidic inlet/outlet interfaces.

Our platform has shown compatibility with testing the intratumor delivery of molecular and cellular therapeutics through the vasculature. Such applications can be further extended to small molecule drugs and nanomedicine, to generate insights into strategies that can enhance the delivery of cancer therapeutics while normalizing the tumor vasculatures in hypoxic tumors. With live-cell imaging and cell tracking, the platform can also be extended to investigate endothelial-immune interaction in cell-based therapies, with a broader set of cell types including natural killer (NK) cells, dendritic cells (DCs), macrophages, engineered T cells, etc. [57]. Furthermore, immunotherapies, particularly those through antigen recognition by T cell receptor (TCR), involve major histocompatibility complex (or human leukocyte antigen, or HLA). An HLA match between immune cell and the targeted cancer cells is crucial for the interpretation of the therapeutic efficacies [58]. The platform developed here only requires a small amount of patient cells but can mimic tumor physiology and potentially reveal therapeutic efficacies in a more faithful way. Thus, it can potentially be applied to incorporate a patient’s own tumor and immune cells to improve personalized cellular therapy.

## Data Availability

All data presented in this manuscript is available upon request to the corresponding author.

## Supplementary Materials

The following are available online at https://www.mdpi.com/article/10.3390/cells10092201/s1, Figure S1: Vascular integrity in endothelial layer, Figure S2: Compaction of vasculature without PDA or fibronectin lining in microfluidic channel.
