# Supplementary material for: Engineering a Vascularized Hypoxic Tumor Model for Therapeutic Assessment
Source: Cells. 2021 Aug 26;10(9):2201. doi: 10.3390/cells10092201 (PMC8468635; doi:10.3390/cells10092201)
Supplement: Supplementary file 1 [file cells-10-02201-s001.zip › cells-1320523-supplementary.pdf]

**Supplemental Materials**

# Engineering a Vascularized Hypoxic Tumor Model for Therapeutic Assessment

Yuta Ando[1], Jeong Min Oh[1], Winfield Zhao[1], Madeleine Tran[1], Keyue Shen[1,2,3*]

[1]Department of Biomedical Engineering, Viterbi School of Engineering, University of Southern California, Los Angeles, CA 90089

[2]Norris Comprehensive Cancer Center, Keck School of Medicine, University of Southern California, Los Angeles, CA 90033

[3]USC Stem Cell, Keck School of Medicine, University of Southern California, Los Angeles, CA 90033

*Correspondence should be addressed to Keyue Shen (keyue.shen@usc.edu)

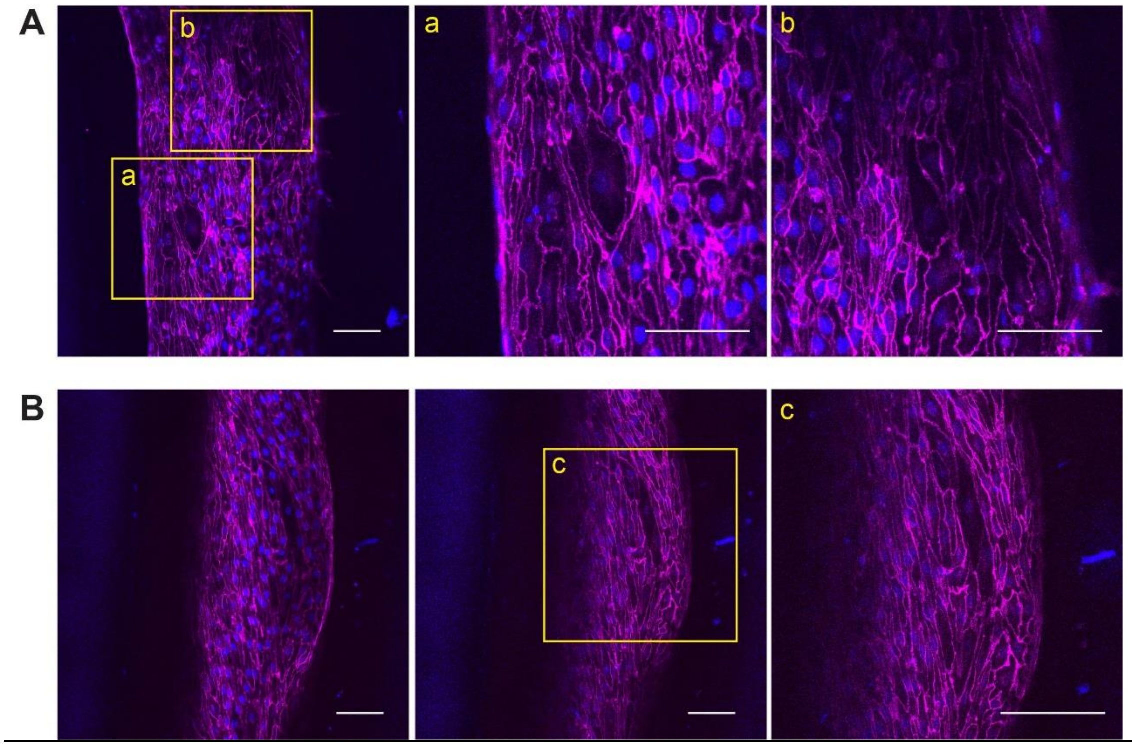


**Supplemental Figure S1.** Vascular integrity in endothelial layer. Purple: VE-cadherin; Blue: nuclei. (**A**) A confocal image taken near the imaging plane of Fig. 3A, and enlargement in the areas a and b. (**B**) Confocal images taken at (left) and near (middle) the imaging plane of Fig. 3B and enlargement in the area c. All images show a continuous layer of endothelium on the vascular wall. Scale bars: 50 μm.

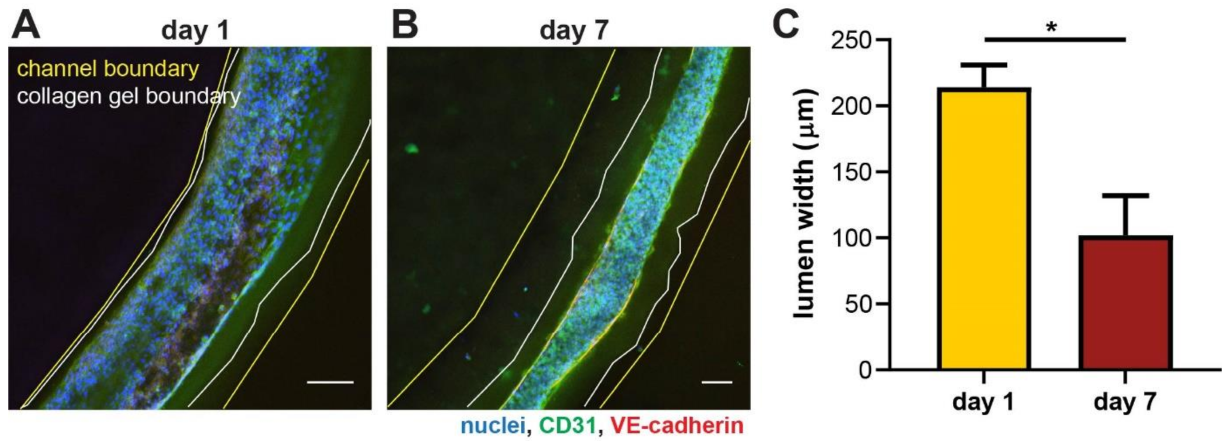


**Supplemental Figure S2.** Compaction of vasculature without PDA or fibronectin lining in microfluidic channel. Vascular morphology/size on (**A**) day 1 and (**B**) day 7. (**C**) Quantitative comparison of the lumen width. Scale bars: 100 μm. *: $p < 0.05$ by $t$-test.
